# Protease inhibitors for the treatment of hepatitis C virus infection

**DOI:** 10.3205/id000034

**Published:** 2017-11-28

**Authors:** Philipp de Leuw, Christoph Stephan

**Affiliations:** 1 Goethe-University Hospital Frankfurt, Medical Clinic II, Infectious Diseases Unit, Frankfurt am Main, Germany

**Keywords:** direct-acting antiviral, hepatitis C, HIV, NS3/4A protease inhibitor

## Abstract

The hepatitis C virus (HCV) has affected an estimate of 80 million individuals worldwide and is a strain of public health. Around 25–30% of patients in Europe and the US infected with HIV are coinfected with HCV. Despite treatment modalities containing a NS3/4A protease inhibitor in combination with pegylated interferon and ribavirin prior to 2013 improved SVR rates, the amount of severe side effects was high. Nowadays, oral direct-acting antivirals (DAAs) combination therapy offers excellent treatment efficacy, safety and tolerability. This review focuses on current literature and clinical evidence and their impact regarding NS3/4A protease inhibitors. In addition, pitfalls in treatment from HIV- and HBV-coinfected patients will also be discussed. In the era of DAA treatment, the third-generation pan-genotypic NS3/4A protease inhibitors (mainly grazoprevir, glecaprevir and voxilaprevir) show a high antiviral activity and genetic resistance barrier with cure rates of over 95% when combined with an NS5A inhibitor, irrespectively of baseline resistance associated variants (RASs) being present. These new key components of DAA combination therapy are impressive options to eradicate HCV in the so called difficult-to-treat population (e.g. compensated cirrhosis, end-stage renal disease and patients who failed previous DAA treatment).

## Introduction

**Figure 1:**
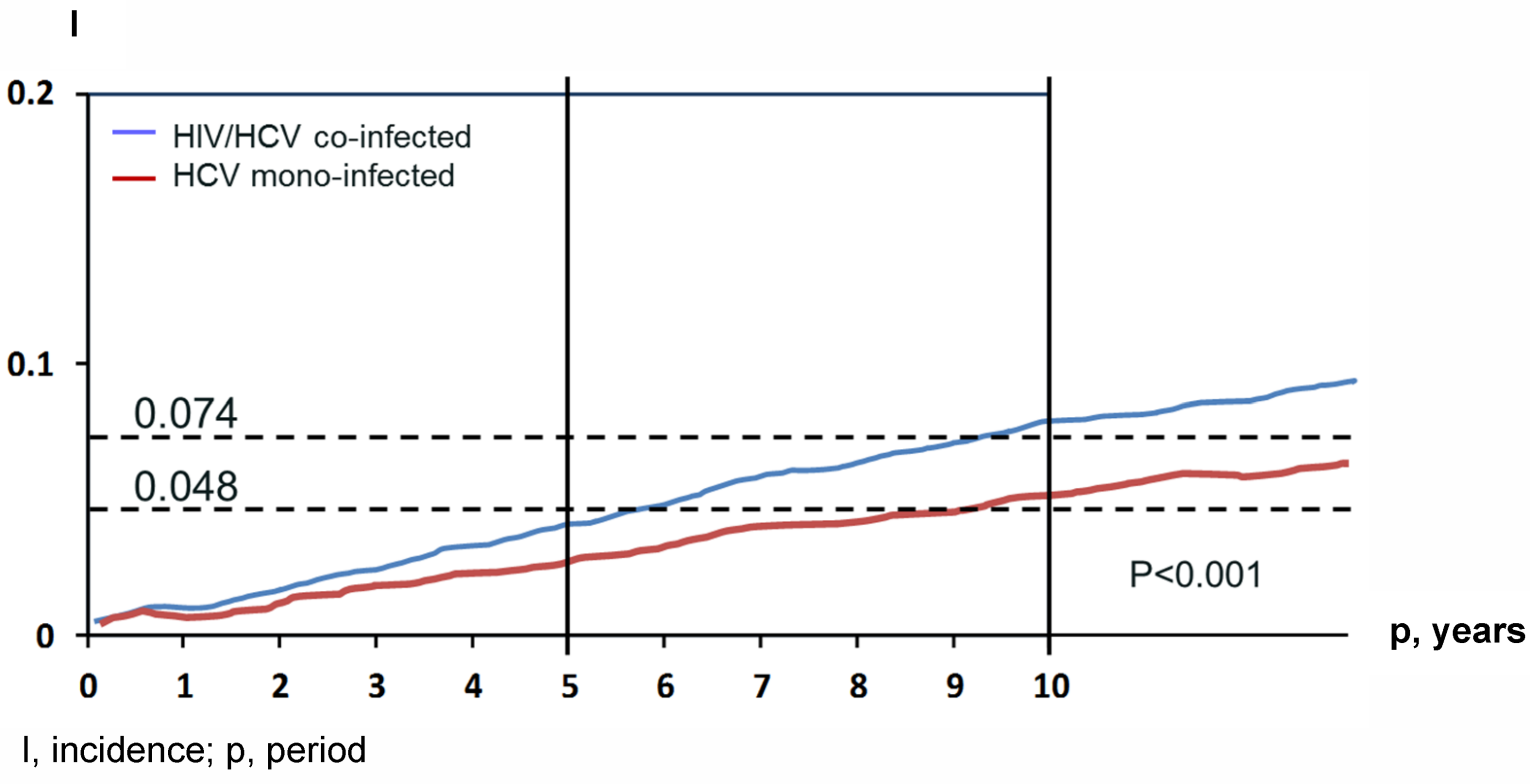
Comparison of the incidence of hepatic decompensation and HCC in HCV/HIV co-infected and HCV mono-infected veterans adapted from Lo et al. 2012 [71]

Approximately 80 million individuals worldwide are estimated to have a chronic hepatitis C virus (HCV) infection [1]. With around 150.000 new cases every year in Western Europe and in the United States, chronic HCV infection is a public health burden [2]. Chronic hepatitis C is strongly associated with the development of cirrhosis, end-stage liver disease and hepatocellular carcinoma (HCC) (Figure 1 (Fig. 1)). Antiviral therapy can prevent these complications [3], [4]. In HIV infected patients, HCV coinfection is a major cause of non-AIDS related morbidity and mortality [5]. Around 25–30% of HIV-positive patients are coinfected with chronic HCV infection in Europe and the USA [6]. In the past decade, standard of care was the administration of interferon (IFN) in association with ribavirin (RBV). Most HCV genotype 1-infected patients achieved in only 40–50% a sustained virological response (SVR) [7], [8], [9] while in genotype 2- or 3-infected patients viral eradication was achieved in ~80% [10]. Since, the development of new antiviral drugs with direct action against the virus, in particular NS3/4A protease inhibitors, started a new era of HCV treatment [11]. Up to now, amazingly high SVR rates of oral direct-acting antivirals (DAAs) combination therapy were reported with an average above 95% at least for genotypes 1 and 4 [12], offering an excellent treatment efficacy, safety and tolerability. These modern all oral therapy consists of a combination of inhibitors of NS5B polymerase, NS3/4A protease and the NS5A replication complex [13], [14]. Protease inhibitors can be generally divided into four sections [13]. This review focuses on protease inhibitors approved in the past 4 years, particularly the second (mainly simeprevir and asunaprevir) and third generation inhibitors (mostly paritaprevir, grazoprevir, glecaprevir and voxilaprevir) will be highlighted. 

## The NS3/4A protease

**Figure 2:**
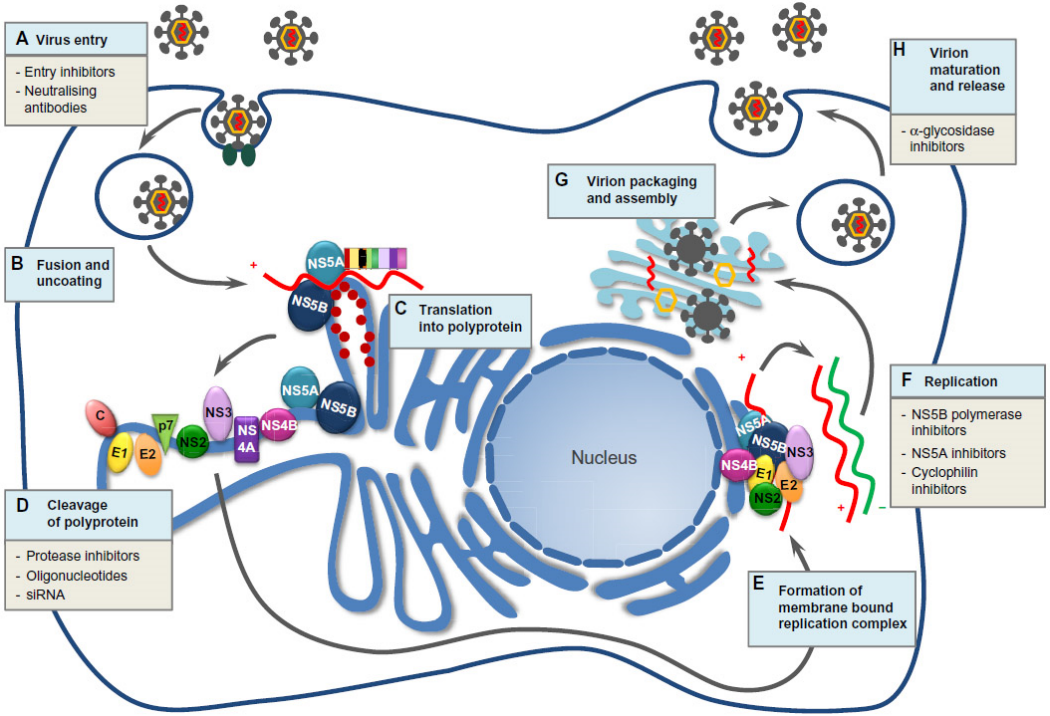
HCV lifecycle and potential targets for DAAs adapted from Holmes et al. 2015 [96] (CC BY-NC 3.0 licence https://creativecommons.org/licenses/by-nc/3.0/)

HCV belongs to the Flaviviridae family and is a small 55 nm virus with a lipid envelope and a single-stranded RNA viral genome with around 9,600 nucleotides [15], [16]. The positive strand RNA genome includes a 5’-non-coding region with an internal ribosome entry site, an open reading frame that encodes structural (core, envelope 1, 2, p7) and non-structural (NS2, NS3, NS4A, NS4B, NS5A, NS5B) proteins and a 3’-non-coding region. The internal ribosome entry site causes the translation of a polyprotein precursor which is processed into mature structural and non-structural proteins [17]. The non-structural NS3-region consists of an N-terminal serine protease and a C-terminal RNA helicase. The NS4A peptide is an important co-factor for the polyprotein maturation. NS4A holds the NS3 protease domain very close to the membrane and serves as a molecular tether that anchors the HCV replication machinery complex together at the cellular membrane [16] (Figure 2 (Fig. 2)). NS4A and NS3 both together form a complex imparting proteolysis of the HCV polyprotein, which splits junctions between the non-structural proteins. The polyprotein itself is required for the replication as well [18]. All NS3 protease inhibitors are linked with the active site of the enzyme [19]. Up-to-date, it is still challenging to design a pan-genotypic NS3 protease inhibitor.

## Resistance to NS3 protease

The high replication rate of HCV generates a heterogeneous virus population in infected patients [20]. The heterogeneity within NS3, NS5A and NS5B areas is a relevant access in the interaction with DAAs. Amino acid polymorphisms associated with suboptimal efficacy of DAAs are denominated as resistance associated substitutions (RASs). RASs can be linked with virological treatment failure. Many RASs are related to a replicative impairment. This is an explanation for the low likeliness of detectable pre-existence RASs as well as a relatively rapid replacement by wild-type virus after quitting protease inhibitor therapy. Despite the low prevalence of NS3 associated RASs in genotype 1 patients (Table 1 (Tab. 1)), there is one important exception. Interestingly, the Q80K has no loss of replicative fitness in numerous patients and is likely to be found with a high prevalence in DAA naïve patients [19]. This variant is linked with different levels of resistance to some protease inhibitors like simprevir, asunaprevir and paritaprevir. Q80K is mainly present in genotype 1a that confers low level resistance to *in vitro* SMV activity [21]. Finally, it is the most common polymorphism among the NS3 RASs linked to low activity of NS3/4A protease inhibitors [22]. Q80K prevalence vary by country with a low overall in genotype 1 infected patients in Europe (up to 19%) compared to North America (48%), respectively [21]. Little is known about RASs for other genotypes. The absence of known RASs for a certain NS3 protease inhibitor does not lead to a high direct-acting antiviral activity. Telaprevir, one of the breakthrough structures, has no impact in genotype 3 patients in a clinical trial, although there were no pre-existing RASs noticed [23]. In conclusion, based on *in vitro* studies, all currently approved NS3 protease inhibitors were designed for HCV genotype 1. The potency against HCV genotype 3 is clearly lower [19]. Table 2 (Tab. 2) shows an overview of the clinical antiviral activity in monotherapy of the recently approved most important HCV protease inhibitors.

## Protease inhibitors

In the course of the past years, four new protease inhibitors have been approved for the treatment of chronic hepatitis C infection (Table 3 (Tab. 3)). 

### Simeprevir (SMV)

Simeprevir has been approved as a once-daily oral NS3/4A protease inhibitor in 2013. The efficacy has been originally established in combination with pegIFN alfa and ribavirin, in HCV genotype 1 infected subjects with compensated liver disease, including cirrhosis [24]. A high antiviral activity was shown in clinical monotherapy trials for SMV in HCV genotype 1, 4 and 6 infected patients. However, activity was low in HCV genotype 2, 3 and 5 patients because of the presence of pre-existing baseline RASs [25], [26]. SMV was the first second-generation protease inhibitor for use in combination with the NS5B polymerase inhibitor sofosbuvir (SOF). Published data from this once-daily regime showed first-time SVR12>90% in genotype 1 patients, irrespectively of ribavirin add-on or the treatment duration (12 weeks vs. 24 weeks) [27]. Finally, these results were a major step forward for the inception of the era of DAA treatment for patients with chronic hepatitis C infection. In the meantime, additional results from two phase 3 trials were published [28], [29]. In the OPTIMIST-1 trial, treatment-naïve and -experienced patients without cirrhosis were randomly allocated to 12 versus 8 weeks of the SMV plus SOF regimen. The SVR 12 rate was 97% versus 83%, respectively. Furthermore, the relapse rate was significantly higher in the 8 weeks’ course. But there was no difference in SVR12 based on genotype 1 subtype or presence of the baseline Q80K RAS [28]. The Optimist-2 trial was designed as a single-arm, open-label investigation for 12 weeks of SMV plus SOF in treatment-naïve or -experienced patients with cirrhosis. Patients without baseline Q80K resistance had similar SVR12 rates, irrespectively from genotype 1 subtype. Nevertheless, patients with subtype 1a infection and the presence of the Q80K RAS had decreased SVR12 rates (74%) [29]. To date, treatment regimens containing SMV are no longer recommended by internatiol and national guidelines. 

### Asunaprevir (ASV)

Asunaprevir is a second generation NS3 protease inhibitor and was initially approved in Japan for use in combination with pegylated IFN and ribavirin. ASV is active against genotype 1, 4, 5 and 6 *in vitro* [30]. The HALLMARK-DUAL trial investigated the safety and efficacy of daclatasvir (DCV), a NS5A inhibitor, once daily plus ASV 100 mg twice daily or placebo for 12 weeks in chronically infected patients with HCV genotype 1b with or without cirrhosis. Even though SVR rates were all-over high in the study population, there were statistically significant lower SVR rates for patients with the presence of pre-existing baseline RASs compared to patients without any baseline RASs (39% versus 92% SVR), respectively. Baseline RASs were detected in 13% of the patients [31]. Overall SVR rates were about 80%. Due to the extensive use of this suboptimal regimen in Japan, DAA failures occurred in approximately 20%. ASV was not approved in the US and Europe. 

### Paritaprevir (PTV)

Paritaprevir is another NS3/4A protease inhibitor (co-dosed with ritonavir (r) as a booster) that was approved for use in combination of the NS5A inhibitor ombitasvir (OBV) and non-nucleoside NS5B polymerase inhibitor dasabuvir (DSV) with or without ribavirin in 2014 [32]. Because of the multitarget antiviral activity, there are important drug-drug interactions, which should be considered before initiating therapy. Furthermore, this combination is not recommendend in patients with Child-Pugh B decompensated cirrhosis or with compensated cirrhosis but with previous episodes of decompensation and with Child-Pugh C decompensated cirrhosis because of the significant higher protease inhibitor concentrations in this difficult-to-treat population [14]. Based on results of major phase III trials, treatment duration of this regimen for patients infected with HCV subtype 1b with or without compensated cirrhosis (without RBV) and subtype 1a without (with RBV) cirrhosis will be 12 weeks. Patients infected with subtype 1a with compensated cirrhosis should receive this combination with ribavirin for 24 weeks [33], [34], [35], [36]. Treatment duration can be shortened from 12 weeks to 8 weeks for patients infected with subtype 1b without cirrhosis, with caution for patients with advanced fibrosis (METAVIR score F3) [37]. In summary, the combination of PTV/r, OBV with DSV with or without RBV is highly potent in patients with chronic HCV infection with genotype 1 with or without cirrhosis. As well, the combination of PTV/r, OBV and RBV without DSV is highly efficacious in patients with HCV genotype 4 infection. In the PEARL-1 and AGATE-1 trials, patients achieved not less than 97% SVR12 rates [38], [39]. Despite the fact that resistance associated polymorphisms will occur fast in monotherapy of the single substance, the combination supplies a high genetic barrier to resistance. Based on a large clinical trial program with more than 2,500 patients, baseline resistance data will be available for nearly 700 patients. None of the patients had RASs within all three agents whereas the prevalence of RASs within one substance was up to 33% in HCV subtype 1a infected patients [40]. The existence of Q80K mutation as a baseline RAS in genotype 1 infected patients showed no difference in the entire group, which is elucidated by the low-level resistance to PTV [21]. However, in the 8-week course SVR12 rates were lower in patients infected with HCV subtype 1a with the pre-existing Q80K mutations (74% vs. 87%), respectively [40], [41]. RASs can be detected in approximately 85% of patients with treatment failure, however NS3 RASs will be observed in only 9% of patients after around 1 year. In especial NS5A and NS5B RASs show a tendency to subsist [42]. 

### Grazoprevir (GZR)

Grazoprevir in combination with the NS5A inhibitor Elbasvir (EBR) was approved for the treatment of HCV infected patients with genotype 1 and 4 by the FDA in 2016 [43]. GZR should be active against all HCV genotypes *in vitro*, but up to now there is only data from experiments performed recently with HCV genotype 1, 2 and 3 available [44]. However, the genetic resistance barrier for the most approved NS3 protease inhibitors is low in monotherapy, a higher barrier was described for GZR caused by the higher antiviral activity against typical RASs emerging in patients with treatment (PTV/r+OBV,DSV +/- RBV) failure, namely R155-, A156- and D168-mutation [26], [45], [46], [47], [48], [49]. Based on pharmacokinetic data from non-HCV infected patients with cirrhosis, the combination of GZR and EBR is contraindicated in patients with Child-Pugh B and Child-Pugh C hepatic impairment. No dose advantage is required in patients with all stages of renal impairment [14]. 

Even though high SVR12 rates (~97%) were across all treatment arms for treatment-naïve and -experienced patients, commonly with genotype 1 and 4 infection, in phase 2 and phase 3 trials [50], [51], [52], [53], [54], [55], there are major restrictions for treatment-naïve and -experienced patients with subtype 1a with or without cirrhosis as well for treatment-experienced genotype 4 patients with or without cirrhosis. Patients in this special population with a HCV RNA level at baseline >800,000 IU/ml should receive the combination for 16 weeks plus RBV if no NS5A resistance test can be performed [14], [43]. Interestingly, RASs in the NS3-region had no impact on SVR. But relevant pre-existing RASs were reported in up to 10% in subtype 1a infected patients. Notably treatment-naïve patients with subtype 1a infection and the presence of baseline RASs had significantly lower SVR rates (22%) compared to patients without baseline RASs (98%), respectively [55]. However, only the prevalence of baseline RASs with a >5fold resistance (M/L28T/A, Q/R30E/H/RG/K/LD, L31M/V/F, H58D, Y93C/H) is relevant in subtype 1a patients [56], [57]. Despite the high antiviral activity of this third-generation protease inhibitor, some major restrictions should be considered. Resistance tests at baseline for pre-existing RASs should be mandatory in patients with HCV subtype 1a whereas there is no necessity in subtype 1b. Recently, promising data were presented for the new 3-drug combination, MK3. MK3 is a three-drug regimen which is formulated into a fixed-dose combination tablet, taken orally twice daily, without regard to food. This triplet consists of the NS5B polymerase inhibitor uprifosbuvir (MK-3682), GZR and the next-generation NS5A inhibitor Ruzasvir (MK-8408). This regimen for 8 or 12 weeks demonstrated high SVR rates (97%) in treatment-naïve patients infected with HCV genotype 1. MK3 was also highly effective in treatment-naïve genotype 2 patients. In conclusion, this triplet regimen is also effective in GT3 treatment-naïve or experienced patients. Additionally, this new combination with RBV add-on and a prolonged treatment duration over 16 weeks offers an option for the very difficult-to-treat population who previously experienced DAA failure with the emergent of NS3 and NS5A RASs [58]. 

### Glecaprevir (GLE, formerly called ABT493)

Glecaprevir is a new pan-genotypic NS3/4A third generation protease inhibitor which was evaluated as a fixed-dose combination with pibrentasvir (PIB, formerly called ABT-530), an NS5A inhibitor. This regimen was approved by the FDA in August 2017. This next generation regimen demonstrated a high barrier to resistance *in vitro* and is potent against the common NS3 variants (e.g. 80, 150, 168) and NS5A variants (e.g. 28, 30, 31 and 93) [59]. In a 3-day monotherapy trial of patients treated with GLE the presence of baseline RASs did not appear to affect the viral load decline during therapy. Patients with HCV subtype 1a and the presence of Q80K at baseline were showing an remarkable decline of viral load [59]. Interestingly, GLE is highly active against wild-type HCV subtype 1a and subtype 1b, as well as subtype 3a. The antiviral activity grants a high level of resistance compared to most second- and third generation protease inhibitors [60]. In part 1 and 2 of the SURVEYOR-I and SURVEYOR-II phase 2 trial, the combination of GLE and PIB without add-on RBV demonstrated high efficacy in patients without cirrhosis across all 6 major HCV genotypes. In patients infected with HCV genotype 1 and 3 with presence of cirrhosis, the fix-dosed regimen with or without RBV achieved SVR rates of 96%–100%. Baseline substitutions had minimal impact on SVR12 rates. The presence of NS5A Y93H mutation at baseline did not affect SVR rates in patients with HCV genotype 3 infection. However, the presence of A30K RAS at baseline increases the risk of treatment failure in genotype 3 infected patients [61], [62]. Patients with HCV genotype 1 or 4 and prior DAA failure were evaluated in the MAGELLAN-1 part 2 trial. They were stratified by HCV genotype and prior DAA experience for receiving 12 or 16 weeks GLE/PIB. The majority had broad representation of baseline NS5A RASs, between 9–11% had dual class resistance. Patients with prior failure to protease inhibitor and NS5A inhibitor containing therapy had significantly lower SVR rates compared to patients with prior failure to PI containing regimen (81% vs. 100%). The presence of NS5A and NS3 RASs at baseline had a strong impact on SVR rates [63]. These findings provide a basis for resistance testing after DAA failure if GLE/PIB is considered as a retreatment option. Nevertheless, this fixed-dose combination represents a major improvement in next generation DAA therapy for treatment-naïve patients and in certain cases as a retreatment option for patients with prior DAA failure. In particular, patients with kidney insufficiency can be easily treated with this once daily regime [64].

### Voxilaprevir (VOX, formerly called GS-9857) 

Voxilaprevir is a NS3/4A protease inhibitor which recently was approved by the FDA as a once-daily single in combination with an NS5B inhibitor, velpatasvir, and an NS5A inhibitor, voxilaprevir (SOF/VEL/VOX) in July 2017. VOX showed potent *in vitro* activity against HCV genotypes 1–6 and an improved resistance profile against frequently genotype 1 NS3 RASs compared to other NS3/4A protease inhibitors, respectively [65]. In a randomized, dose ranging phase 1 study, VOX demonstrated a potent antiviral activity with or without the presence of commonly observed NS3 mutations with resistance to protease inhibitors [66]. The New Drug Application (NDA) is based on data from the POLARIS-1 phase 3 trial and the POLARIS-4 phase 3 trial, which reported high SVR rates (97%) in patients with HCV genotypes 1–6, including those who failed to prior treatment with an NS5A-containing regimen. The POLARIS-1 study evaluated the treatment combination of SOF, VEL and VOX as a fixed dose single-tablet regime for 12 weeks in patients who previously received an NS5A inhibitor. Overall high SVR rates (96%) were shown in this difficult-to-treat population. Among the group of relapsers, no treatment emergent RASs were reported. Six relapses were observed in patients with cirrhosis at baseline, no relapse in patients without any clinical signs of cirrhosis. Latterly presented data from the POLARIS-4 trial showed formidable high SVR12 rates (97%) in DAA experienced patients infected with HCV genotype 1–3 with or without cirrhosis who had not previously been treated with an NS5A inhibitor. Baseline RASs did not affect treatment outcome. Impressively, no treatment RASs emerged in patients treated with SOF/VEL/VOX [67]. The NDA is further supported by two additional phase 3 studies (POLARIS-2 and POLARIS-3) in which DAA-naïve HCV-infected patients received 8 weeks of SOF/VEL/VOX. The so-called POLARIS 2-trial compared the treatment with the single-tablet combination of SOF, VEL and VOX for 8 weeks to treatment with SOF and VEL for 12 weeks in patients infected with HCV genotype 1–6 with and without cirrhosis who have not previously been treated with a DAA. Treatment with the single-tablet regimen resulted in high SVR rates in genotype 1–6 DAA-naïve patients. But higher relapse rates were reported for this combination in the 8 weeks’ arm compared to the arm being treated with SOF and VEL for 12 weeks, particularly in HCV subtype 1a patients with the presence of Q80K RAS at baseline. Despite the fact of high susceptibility from Q80K *in vitro* to VOX, patients who received SOF/VEL/VOX for 8 weeks had significant lower SVR rates with the presence of Q80K (88% vs. 94%). Data from the POLARIS-3 trial reported high SVR rates (96%) in patients infected with HCV genotype 3 and cirrhosis, irrespectively from the existence of Y93H at baseline. Interestingly, no treatment associated RASs were discovered in the SOF/VEL/VOX arm whereas in the SOF/VEL arm failures evolved an Y93H. The most common adverse events among patients who received SOF/VEL/VOX were headache, fatigue, diarrhea and nausea [68]. In conclusion, lower relapse rates can be expected for this single-tablet 3-drug regimen compared to the combination of SOF and VEL. Up to date, there is no distinct evidence if this regimen is superior to the dual combination of GLE and PIB in treatment naïve patients. However, this fixed-dosed regimen will become important in salvage therapy and in difficult-to-treat treatment-naïve patients. 

## HIV/HCV coinfection

While AIDS-related mortality has decreased, liver related mortality due to HCV coinfection has emerged as a major cause for non-AIDS associated morbidity and mortality [69], [70]. Data from the Veterans Aging Cohort Study Virtual Cohort showed a higher risk of hepatic decompensation and death in HIV coinfected patients compared to HCV-monoinfected individuals. Notably the risk of decompensation was higher for coinfected patients with advanced liver fibrosis, severe anemia and non-black race [71]. Consequently, the recently published and updated EASL and AASLD guidelines highly recommend treating all HCV coinfection patients with prioritization. HIV-coinfected patients should be treated and retreated the same as patients without HIV infection [14], [72], [73]. However, there are a couple of restrictions in consequence of drug-drug interaction between the combined antiretroviral therapy (cART) and DAAs. Especially NS3/4A protease inhibitors interact with boosters like ritonavir and cobistat, HIV protease inhibitors (PI), non-nucleoside reverse-transcriptase inhibitors (NNRTIs) and entry inhibitors (most notably maraviroc). A switch of the cART before starting HCV treatment with DAAs is mainly forced by PTV/r, SMV and GZR in most patients. Nevertheless, since integrase inhibitors containing cART regimens are recommended as a first-line therapy, a favorable interaction profile is increasing. Dose adjustments are necessary if the NS5A inhibitor DCV is coadministrated with ritonavir-boosted atazanavir or efavirenz. Table 4 (Tab. 4) summarizes and highlights the most important drug-drug interactions. For additional drug-drug interactions, data the interaction checker from the university of Liverpool represents a very well established online tool (http://www.hep-druginteractions.org), also available in app format (Liverpool HEP iChart). Real-life cohorts evaluated the safety and efficacy of DAAs in HIV coinfected patients. A lately presented prospective multicohort study from Spain demonstrated a worse respond to DAA-based therapy in HIV-coinfected patients compared to HCV-monoinfected patients (95 vs. 97%), respectively [74]. The underlying reason of this major finding was unclear. However, encouraging SVR12 rates were shown for HIV-coinfected individuals in the German Hepatitis C-registry. At baseline, cirrhosis was less frequent in HIV-coinfected patients and the majority was male. Overall, there were no differences in SVR rates between HIV-coinfected and HCV-monoinfected patients [75]. In summary, these recently presented data give strong evidence that there is no need to recommend different treatment options and regimens for HIV-coinfected individuals. Nonetheless, there is one important exception. In the setting of acute HCV infection, SVR rates were significantly lower in patients with HIV-coinfection (77%, 20/26), who received a fixed-dose combination of SOF and LDV, compared to HCV-monoinfected patients (100%, 20/20), respectively [5], [6]. To date, all DAA regimens are not approved for treatment in patients with acute HCV-infection. Furthermore, there are still unacknowledged questions such as the relevance of transmitted RASs in the era of DAA treatment. 

## HBV/HCV coinfection

Even though the prevalence of hepatitis B virus (HBV) coinfection varies, there are populations at high risk for acquiring both infections due to common routes of transmission. The prevalence of HBV/HCV coinfection in two US Veterans Administration cohorts was 42–67% [76]. Lately, the FDA warns about the risk of HBV becoming an active infection again in any patient who has a current or previous infection with HBV and is treated with certain DAA medicines for hepatitis C virus [77]. HBV reactivation is defined as the increase in HBV-DNA in a patient with inactive or resolved HBV infection. Reactivation can occur spontaneously, but is mostly triggered by immunodeficiency due to HIV or immunosuppressive therapy. In total, 29 cases were collected globally. Interestingly, HBV reactivation can occur early after starting DAA therapy, the median time to occurrence of reactivation was 46 days. Three patients developed decompensated liver failure. There was no HCV genotype or drug specific risk but rather a general risk for HBV reactivation under DAA therapy. Most patients with HBV reactivation were patients with known chronic HBV infection and a positive HBs-Antigen. However, HBV reactivation has also been reported in HBV/HCV coinfected patients treated with IFN-based HCV therapy. But compared to patients under IFN based therapy, development of hepatitis was more likely in DAA treated patients [78]. The mechanism through which HBV reactivation occurs with DAAs is currently unknown. In a prospective, multicenter cohort at 14 sites in Taiwan patients with chronic HBV/HCV coinfection treated with LDV/SOF for 12 weeks were observed for HBV reactivation. Treatment with LDV/SOF was associated with silent HBV viral reactivation in 63% of patients (70/111). Nevertheless, no patient experienced clinical signs or symptoms of HBV reactivation [79]. Results from a retrospective analysis of HBV reactivation in 62,920 American veterans showed varying severety of reactivation. However, the clinical presence of liver failure was rare [80]. In conclusion, HBV screening is mandatory for all HCV patients before initiating treatment with DAAs. Especially patients with anti-HBc should be monitored more closely during DAA treatment and post-treatment follow-up. Lately, the updated EASL guideline recommends nucleos(t)ide analogue prophylaxis for HBsAg-positive patients undergoing DAA therapy until week 12 post DAA [81]. 

## Conclusion

Due to high antiviral activity and high genetic resistance barrier NS3/4A protease inhibitors are an important element of HCV treatment. In combination with DAAs of one or two other classes they can stop different stages of viral life cycle and therefore they can achieve SVR rates above 95% [12]. Protease inhibitors are well tolerated and have a low incidence of side effects, also in patients with renal impairment and compensated cirrhosis. HCV infected patients with genotype 3 represent up to 30% of all HCV infections worldwide. In past years of DAA treatment they belong to the difficult-to-treat subgroup [82]. Numerous NS3/4A protease inhibitors exhibit significantly lower potency against HCV genotype 3, caused by polymorphisms between genotypes in the drug target altering the intermolecular dynamics of the protein-inhibitor complex [83]. But reported results from new treatment combinations of pan-genotypic protease inhibitors, namely GZR, GLE and VOX, look promising and showed high SVR rates in this difficult-to-treat population irrespectively in the most cases from baseline RASs [60], [61], [63], [66], [67], [68], [64], [62]. A baseline resistance test was recommended from experts to patients receiving a treatment combination of a second-generation protease inhibitor, mainly SMV, and a NS5A-inhibitor with a relative low barrier to resistance. To date, in the era of new pan-genotypic third generation NS3/4A protease inhibitors, the presence of baseline RASs or even the onset of RASs during treatment does not affect SVR rates in most cases at all. But however, there is still a demand of carefulness, because SVR rates were significantly lower in HCV genotype 1a patients receiving SOF/VEL/VOX for 8 weeks with the presence of Q80K RAS at baseline and in HCV genotype 3 patients with NS5A baseline RASs who received GLE/PIB. Anyway, these regimens are excellent tools to treat patients who failed previous treatment with DAAs, including therapy with an NS5A-inhibitor or NS3/4A protease inhibitor. These combination therapies should be evaluated in a real-life setting and trials need to validate whether resistance testing prior therapy is clinically relevant or not. 

However, guidelines recommend genotype testing prior to HCV treatment initiation [14], [72], testing is very expensive and is not widely available in low and middle-income countries because it requires advanced equipment. The new DAA combinations with a pan-genotypic third generation NS3/4A protease inhibitor represent an opportunity to treat HCV infected patients without prior genotype testing in low and middle-income countries. Finally, diagnostic simplification and cost-reduction will be the key to enable implementation of HCV screening and treatment in those countries [84]. Current prices of these regimens are variable and unaffordable globally. Hopefully, this situation will improve in future and these novel third generation NS3/4A protease inhibitors will be accessible for all HCV infected patients. 

Summing up, the new third generation NS3/4A protease inhibitors in combination with other DAAs can be a substantial step in the eradication of HCV in the so called difficult-to-treat population (e.g. compensated cirrhosis, end-stage renal disease and patients who failed previous DAA treatment). Anyway, a real decrease of HCV infections in global population demands available HCV testing in low-income countries, access to health care and treatment. All together we should ensure that these lifesaving treatments become accessible to all those who need them. 

## Abbreviations

- 3D: ritonavir-boosted paritaprevir, plus ombitasvir and dasabuvir
- ASV: asunaprevir
- cART: combined antiretroviral therapy
- BOC: boceprevir
- DAA: direct-acting antiviral
- DCV: daclatasvir
- DSV: dasabuvir
- EBR: elbasvir
- GLE: glecaprevir
- HBV: hepatitis B virus
- HCV: hepatitis C virus
- HCC: hepatocellular carcinoma
- IFN: interferon
- LDV: ledipasvir
- NDA: new drug application
- NNRTI: non-nucleoside reverse-transcriptase inhibitor
- OBV: ombitasvir
- PIB: pibrentrasvir
- PTV: paritaprevir
- r: ritonavir
- RAS: resistance associated substitutions
- RBV: ribavirin
- SMV: simeprevir
- SOF: sofosbuvir
- SVR: sustained virological response
- TVR: telaprevir
- VEL: velpatasvir
- VOX: voxilaprevir

## Notes

### Competing interests

- **de Leuw P, M.D.,** Specialist for Internal Medicine & Infect. Diseases
  Consultancies/speaker’s bureau/travel support for Bristol-Myers Squibb GmbH & Co. KGaA, Gilead Sciences GmbH, GlaxoSmithKline GmbH & Co.KG, Hexal AG, MSD Sharp & Dohme GmbH, Janssen-Cilag GmbH
- **Stephan C, M.D.,** Professor for Internal Medicine & Infect. Diseases Consultant
  - Receipt of grants/research supports: MSD
  - Receipt of honoraria or consultation fees (any): AbbVie, MSD, ViiV, BMS, Gilead, Janssen, Astellas, Stada
  - Receipt of travel grants within last 2 years: Janssen, Gilead Sciences, BMS

## Figures and Tables

**Table 1:**
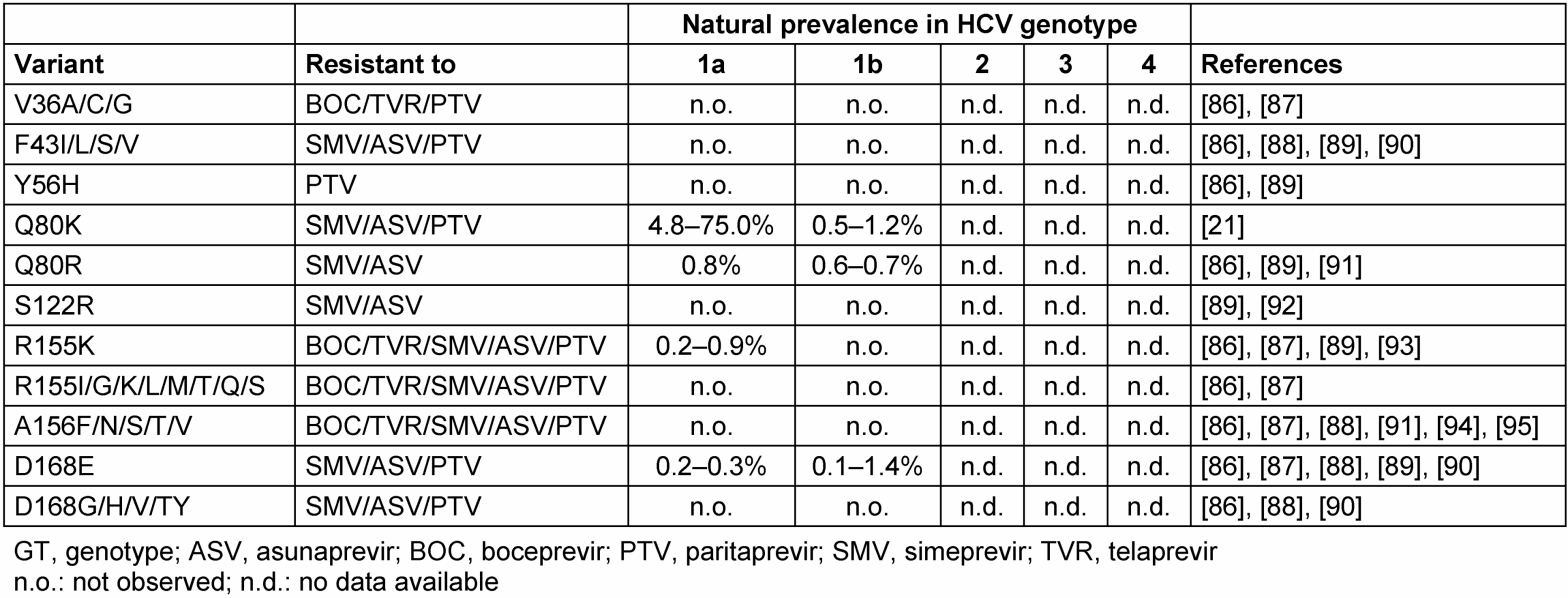
Natural prevalence of NS3 protease-inhibitors associated RAVs detected by population sequencing adapted from Sarrazin 2016 [19]

**Table 2:**
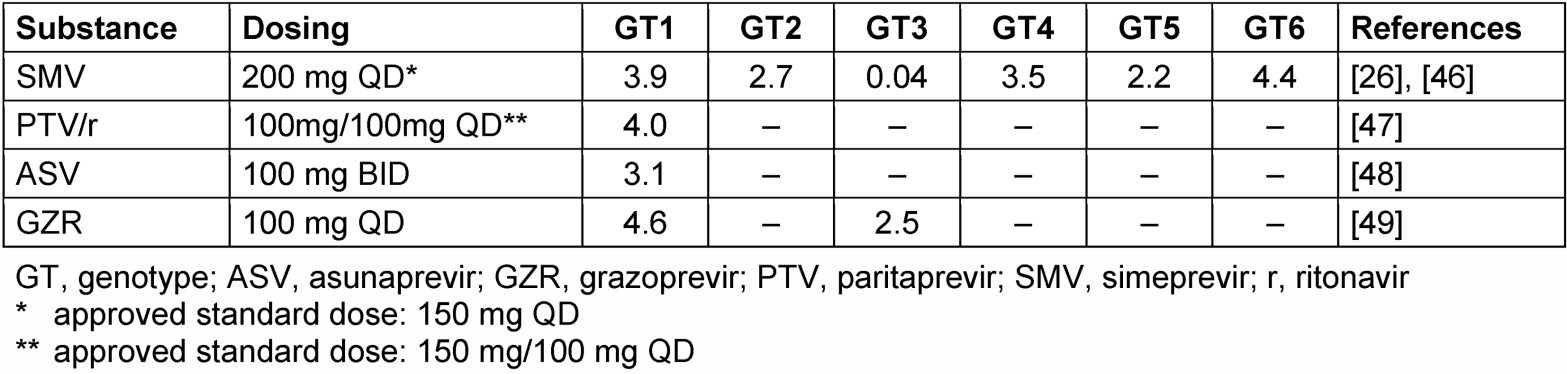
Clinical antiviral activity of the most important approved protease inhibitors (mean or median maximum HCV viral load decline after 3–14 days’ monotherapy [log10 IU/ml] adapted from Sarrazin 2016 [19]

**Table 3:**
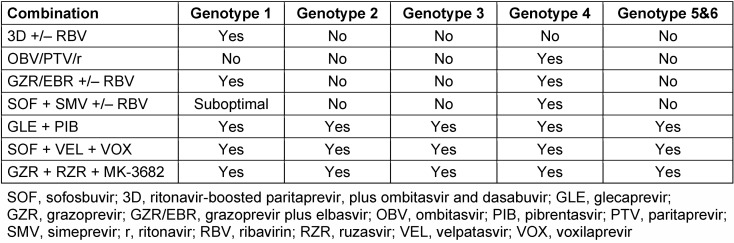
NS3/4A protease inhibitors containing IFN-free combination treatment regimens and their treatment recommendations as per genotype adapted from EASL Recommendations on Treatment of Hepatitis C 2016 [14]

**Table 4:**
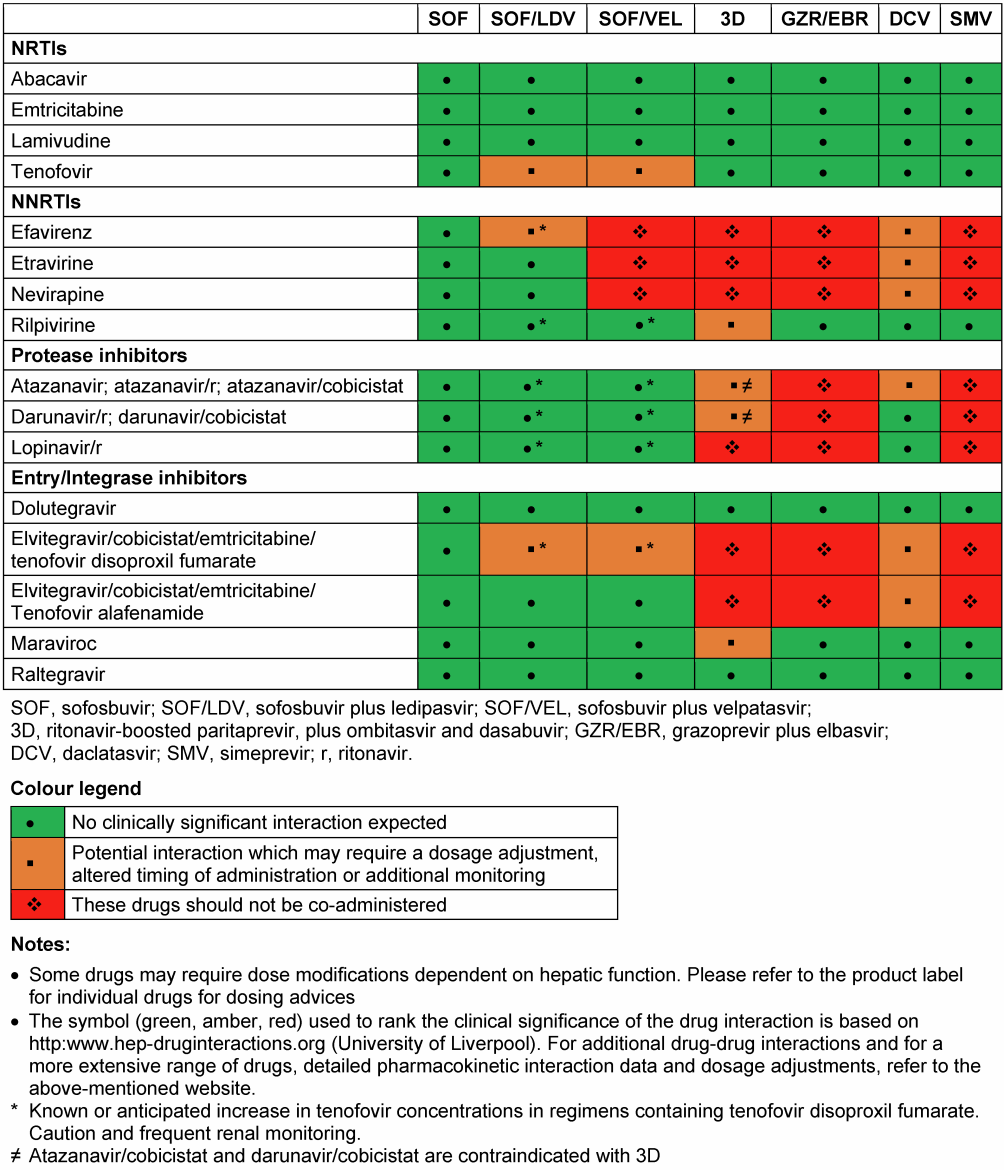
Drug interactions between HCV DAAs and HIV antiretrovirals adapted from EASL Recommendations on Treatment of Hepatitis C 2016 [14]
